# Hipertensão Arterial Pulmonar com Características de Envolvimento Venoso: Um Trabalho Investigativo

**DOI:** 10.36660/abc.20230565

**Published:** 2024-04-15

**Authors:** Daniel Inácio Cazeiro, Rui Plácido, Miguel Azeredo Raposo, Joana Brito, Alexandra Borba, Tatiana Guimarães, Eugénia Pinto, Pedro Freitas, Fausto J. Pinto

**Affiliations:** 1 Universidade de Lisboa Faculdade de Medicina de Lisboa Centro Hospitalar Universitário Lisboa Norte Lisboa Portugal Departamento de Coração e Vasos – Centro Hospitalar Universitário Lisboa Norte – Faculdade de Medicina de Lisboa – Universidade de Lisboa, Lisboa – Portugal; 2 Centro Hospitalar Universitário Lisboa Ocidental Serviço de Cardiologia Carnaxide Portugal Serviço de Cardiologia – Centro Hospitalar Universitário Lisboa Ocidental, Carnaxide – Portugal; 3 Centro Hospitalar Universitário Lisboa Central Serviço de Pneumologia Lisboa Portugal Serviço de Pneumologia – Centro Hospitalar Universitário Lisboa Central, Lisboa – Portugal; 4 Centro Hospitalar Universitário Lisboa Central Serviço de Patologia Lisboa Portugal Serviço de Patologia – Centro Hospitalar Universitário Lisboa Central, Lisboa – Portugal

**Keywords:** Hipertensão Arterial Pulmonar, Doença Veno-Oclusiva Pulmonar, Transplante de Pulmão, Hemangioma Capilar/cirurgia, Tomografia Computadorizada/diagnóstico por imagem

## Abstract

A doença veno-oclusiva pulmonar (DVOP) e a hemangiomatose capilar pulmonar são tipos raros de substratos histopatológicos dentro do espectro da hipertensão arterial pulmonar (HAP) com prognóstico muito ruim. Caracterizam-se por um processo fibroproliferativo generalizado das veias e/ou capilares de pequeno calibre com preservação das veias maiores, resultando em um fenótipo de hipertensão pulmonar pré-capilar. A apresentação clínica é inespecífica e semelhante a outras etiologias de HAP. O diagnóstico definitivo é obtido por meio de análise histológica, embora a biópsia pulmonar não seja aconselhada devido ao maior risco de complicações. No entanto, alguns achados adicionais podem permitir um diagnóstico clínico presuntivo de DVOP, especialmente história de tabagismo, uso de drogas quimioterápicas, exposição a solventes orgânicos (particularmente tricloroetileno), baixa capacidade de difusão do monóxido de carbono (DLCO), dessaturação ao esforço e evidências de doença venosa sem doença cardíaca esquerda no exame de imagem, manifestada por uma tríade clássica de opacidades em vidro fosco, linhas septais, e linfadenopatias. O transplante pulmonar é o único tratamento eficaz e os pacientes devem ser encaminhados no momento do diagnóstico, devido à rápida progressão da doença e ao prognóstico ruim.

Apresentamos o caso de um homem de 58 anos com HAP com características de envolvimento venoso/capilar em que a suspeita clínica, o pronto diagnóstico e o encaminhamento precoce para transplante pulmonar foram determinantes para um bom desfecho.

## Introdução

### Definição, Epidemiologia e Fatores de Risco

A doença veno-oclusiva pulmonar (DVOP) e a hemangiomatose capilar pulmonar (HCP) são consideradas parte do espectro da mesma doença e classificadas como um subgrupo da hipertensão arterial pulmonar (HAP) devido às suas semelhanças clínicas, histopatológicas e hemodinâmicas.

A DVOP representa uma minoria de pacientes com hipertensão pulmonar (HP), representando cerca de 10% dos casos de HAP, com incidência anual estimada de 0,1 a 0,2 casos por milhão.^1 , 2^ Entretanto, devido à classificação incorreta de alguns casos como HAP idiopática ou HP tromboembólica crônica, a verdadeira incidência a doença pode estar subestimada.^3 – 5^ A DVOP tende a acometer homens e mulheres na mesma proporção,^6^ embora pareça haver predomínio masculino nos casos idiopáticos.^7^

Apesar de sua patogênese desconhecida, diversos fatores de risco já foram identificados para a DVOP. Mutações bialélicas do gene EIF2AK4 foram reconhecidas em casos familiares e em cerca de 25% dos casos esporádicos.^8 , 9^ A exposição a alguns quimioterápicos, como bleomicina, mitomicina, cisplatina, entre outros,^10 – 13^ e a solventes orgânicos (principalmente tricloroetileno)^7^ está associada a maior risco. Em comparação com outros subtipos de HAP, pacientes com DVOP apresentam maior exposição prévia ao tabagismo.^6^ Parece haver uma associação com doenças autoimunes, principalmente esclerose sistêmica.^14 , 15^ Diversos outros mecanismos foram propostos, mas provavelmente não são causadores (infecções, diátese trombótica, fatores congênitos e contraceptivos orais).^16^

### Histopatologia

A DVOP é uma doença fibroproliferativa que afeta principalmente as pequenas veias pulmonares, com relativa preservação das veias maiores. Há oclusão extensa e difusa das veias de pequeno calibre devido à hipertrofia da musculatura lisa e deposição da matriz de colágeno.^1 , 17^ Lesão venosa de longa duração pode resultar em arterialização das veias pulmonares devido ao aumento das fibras elásticas, muitas vezes confundida com alterações da HAP.^1^

Pode haver o envolvimento concomitante das arteríolas pulmonares, com características histológicas comuns à HAP. Contudo, geralmente não há lesões plexiformes arteriais (típicas na HAP).^1 , 18^ Os capilares alveolares podem estar ingurgitados, consistente com HCP. Um estudo de coorte retrospectivo revelou que 73% dos pacientes com DVOP apresentavam HCP coexistente, ao passo que a maioria dos pacientes que receberam diagnóstico de HCP também apresentava alterações venosas, postulando a hipótese de que a DVOP e a HCP representam o mesmo diagnóstico.^17^

Outros achados incluem dilatação dos vasos linfáticos pulmonares/pleurais, provavelmente secundária à congestão venosa,^18^ e macrófagos alveolares contendo hemossiderina, como resultado de congestão crônica e hemorragia.^19^

### Avaliação clínica e diagnóstico

O diagnóstico definitivo de DVOP é obtido por meio de exame histopatológico do tecido pulmonar. Entretanto, a biópsia não é recomendada devido ao risco aumentado de sangramento pulmonar com risco de vida.^1^ Portanto, a confirmação histológica pode não ser obtida até que seja analisado um pulmão explantado ou realizada uma autópsia. Atualmente, recomenda-se uma abordagem diagnóstica não invasiva baseada em determinadas características clínicas e resultados de exames diagnósticos.^6^

A apresentação clínica da DVOP é inespecífica e semelhante a outras etiologias de HAP.^6^ De modo semelhante, não há diferenças hemodinâmicas significativas.^6^ Pacientes com DVOP apresentam pressão em cunha normal paradoxal pelo fato de a doença ocorrer nas pequenas veias, sem obstrução das veias pulmonares maiores. Portanto, a coluna estática de sangue produzida pelo cateter reflete a pressão normal nas veias maiores, com o mesmo calibre do vaso ocluído.^6 , 20^ Em outras palavras, a pressão arterial pulmonar em cunha (PAPC) mede as pressões nas veias pulmonares maiores que não são afetadas pela doença e não as pressões reais aumentadas nas pequenas vênulas e capilares pulmonares. No entanto, alguns achados adicionais podem levantar suspeita para um diagnóstico de DVOP:

– Evidências de congestão venosa sem doença cardíaca esquerda significativa podem ser observadas em imagens do tórax de pacientes com DVOP, geralmente na forma de opacidades difusas em vidro fosco, espessamento septal e linfadenopatia mediastinal.^6 , 21^ Em um estudo, quando um ou mais desses três elementos estava presente, a sensibilidade e especificidade foram de mais de 80 e 67 por cento, respectivamente.^21^ Outro estudo com 25 pacientes relatou a presença de pelo menos uma característica radiológica típica em todos os pacientes e pelo menos dois achados radiológicos em 92% deles.^22^ Entretanto, a ausência ou a presença de apenas um sinal na tomografia de alta resolução não exclui completamente o diagnóstico de DVOP. Se houver incerteza quanto ao diagnóstico de DVOP, pode ser útil repetir a tomografia de alta resolução durante o curso da doença, pois pode revelar piora progressiva das anormalidades, principalmente após o início do tratamento.
– Os testes de função pulmonar (TFP) e o teste de caminhada de seis minutos (TC6) também são muito importantes para ajudar a distinguir entre DVOP e HAP. Pacientes com DVOP apresentam DLCO muito mais baixa, bem como saturação mínima de oxigênio no TC6;^6^
– Desenvolvimento de edema pulmonar após teste de vasorreatividade aguda ou administração de vasodilatadores pulmonares, provavelmente devido ao aumento da pressão hidrostática transcapilar, a qual causa a transudação de líquido para o espaço alveolar.^6 , 23 , 24^ Esta característica é considerada altamente sugestiva de DVOP. Pacientes com resposta vasodilatadora aguda podem desenvolver edema pulmonar grave rapidamente após o início da terapia com bloqueadores dos canais de cálcio e este tratamento é contraindicado.

A combinação de HP pré-capilar por meio de cateterismo cardíaco direito [pressão arterial pulmonar média (PAPm) > 20 mmHg, PAPC ≤ 15 e resistência vascular pulmonar (RVP) > 2 unidades Wood], os achados acima e a presença de fatores de risco reforçam a diagnóstico clínico de DVOP.^2^

### Manejo

O transplante pulmonar bilateral é atualmente considerado a única terapia eficaz para DVOP.^1 , 2 , 16^ Devido à rápida progressão da doença, os pacientes devem ser encaminhados no momento do diagnóstico.^1 , 2 , 16^

As medidas de suporte incluem oxigenoterapia, diuréticos, vacinas antipneumocócicas, contra influenza e contra o SARS-CoV-2, bem como cessação do tabagismo. A anticoagulação é controversa. Ao passo que alguns estudos observacionais sugerem benefício, outros não aconselham devido à maior frequência de hemorragia alveolar oculta.^25^

A terapia específica para HP pode ser administrada como uma ponte para o transplante pulmonar.^2^ Entretanto, os pacientes apresentam risco aumentado de desenvolver edema pulmonar, insuficiência respiratória e até morte.^23^ Quando indicado, recomenda-se um ensaio cauteloso com um único agente oral (inibidores da fosfodiesterase ou antagonistas dos receptores da endotelina) em pacientes sintomáticos com aumento gradual da titulação e monitoramento rigoroso de complicações. Em pacientes mais graves, o tratamento intravenoso com epoprostenol também pode ser considerado.^2^

### Prognóstico

O prognóstico da DVOP é ruim. Estudos relatam mortalidade de até 72% em um ano.^23^ O tempo médio desde os primeiros sintomas até a morte ou transplante pulmonar é de dois anos.^6^

### Caso Clínico

Um homem de 58 anos foi encaminhado à consulta ambulatorial devido à história de dispneia ao esforço rapidamente progressiva (classe funcional III da OMS), tosse, síncope por esforço e edema de membros inferiores há dois meses.

Apresentava diagnóstico prévio de enfisema pulmonar, sem comprometimento funcional, atribuído a tabagismo anterior (40 anos-maço) e dislipidemia. Devido ao seu trabalho anterior como técnico de aviação, o paciente havia sido exposto a diversos agentes, como compostos fenólicos, resinas e fibras de vidro.

O paciente estava em uso de inalador de brometo de umeclidínio/vilanterol (55/22 ug uma vez ao dia), furosemida (40 mg uma vez ao dia) e estazolam (2 mg duas vezes ao dia). Também estava recebendo oxigenoterapia há um mês.

Negou qualquer história familiar prévia de HP.

**Figura 1:**
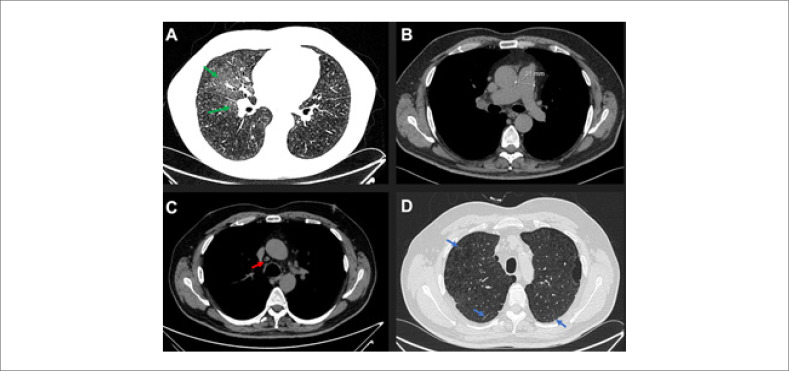
Características da doença veno-oclusiva pulmonar na tomografia computadorizada de alta resolução. Painel A – opacidades centrolobulares em vidro fosco (setas verdes). Painel B – tronco principal pulmonar dilatado. Painel C – linfonodomegalia látero-aórtica (seta vermelha). Painel D – presença de linhas septais (setas azuis).

O paciente foi submetido a diversos exames diagnósticos:

- Os exames laboratoriais mostraram eritrocitose (nível de hemoglobina de 17,7 g/dL) e NT-proBNP elevado (3110 pg/mL). A investigação laboratorial básica de imunologia, incluindo testes de triagem para anticorpos antinucleares, anticorpos anticentrômero e anti-Ro, não apresentou alterações dignas de nota.
- O eletrocardiograma (ECG) revelou desvio elétrico do eixo direito do QRS no plano frontal, bloqueio incompleto de ramo e padrão de sobrecarga ventricular direita.
- O ecocardiograma transtorácico demonstrou alta probabilidade de HP com cavidades cardíacas direitas dilatadas e função sistólica do ventrículo direito prejudicada. Além disso, excluiu doença cardíaca esquerda.
- O TFP mostrou DLCO muito baixa (35%) mesmo quando corrigido para volume alveolar (45%) com volumes e fluxos preservados.
- Distância de 180 metros no TC6 e dessaturação significativa mediante esforço (nadir de 79%) sem oxigênio suplementar.
- A angiotomografia computadorizada (ATC) mostrou alterações enfisematosas, opacidades em vidro fosco e linfadenopatias mediastinais, sem sinais de doença tromboembólica ( Figura 1 ).
- O cateterismo cardíaco direito confirmou o diagnóstico de fenótipo pré-capilar, com pressão média da artéria pulmonar de 45 mmHg, pressão de oclusão capilar pulmonar de 15 mmHg, resistência vascular pulmonar de 7,5-8,5 unidades Wood e índice cardíaco de 1,98 L/min/m2. O teste de vasorreatividade aguda realizado com óxido nítrico inalado a 30 ppm foi negativo e sem complicações.
- Os testes genéticos com painel de sequenciação de próxima geração excluíram quaisquer mutações relevantes, especificamente no gene EIF2AK4.

Considerando o histórico prévio de tabagismo, rápida progressão dos sintomas, DLCO muito baixa na ausência de doença pulmonar parenquimatosa significativa e achados radiológicos na ATC, presumiu-se o diagnóstico de HAP com características de envolvimento venoso/capilar (Grupo 1.6). Neste cenário clínico, foi realizada terapia combinada sequencial inicial com tadalafil e bosentana e o paciente foi encaminhado com urgência para transplante pulmonar bilateral.

Durante o seguimento, após cuidadoso aumento da titulação da terapia vasodilatadora e diurética, oxigênio suplementar e inclusão em programa de reabilitação cardiorrespiratória, houve melhora na distância percorrida no TC6 (180 ➩ 300 m), na saturação de O_2_ mediante esforço (nadir de 92%) e nos níveis de NT-proBNP (3310 ➩ 1382 pg/mL).

**Figura 2:**
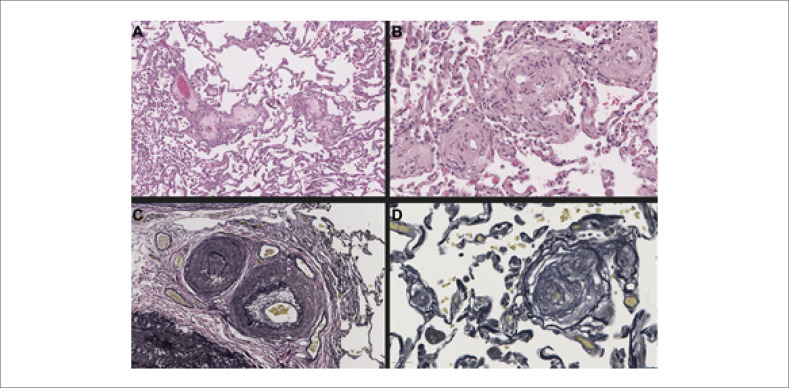
Histologia dos pulmões do paciente. Painel A – Coloração com hematoxilina-eosina mostrando veias septais e vênulas pré-septais obliteradas por fibrose frouxa rica em colágeno; fora isso, o parênquima não apresenta remodelação intersticial importante. Painel B – Coloração com hematoxilina-eosina mostrando focos irregulares de hemangiomatose capilar em associação com vasos e microvasos pulmonares remodelados (centro). Painéis C e D – Colorações de Verhoeff mostrando hipertrofia e hiperplasia intimal fibrosa da camada muscular com suboclusão dos lúmens. Escalas: A), C) 100 μm; B) 50 μm; D) 20 μm.

O paciente foi submetido a transplante pulmonar bilateral seis meses após a primeira consulta e nove meses após o início dos sintomas. A histopatologia dos pulmões explantados revelou oclusão parcial/total de veias de pequeno calibre devido à proliferação fibrosa intimal bem como oclusão de artérias de pequeno calibre com hiperplasia intimal, hipertrofia medial e recanalização, fibrose de septos alveolares, congestão vascular, áreas de edema e hemorragia, bem como descamação pigmentada de macrófagos nos espaços alveolares ( Figura 2 ). Esses achados confirmaram o diagnóstico de HAP com características de envolvimento venoso/capilar.

As características clínicas gerais deste caso estão de acordo com o que foi publicado em diversas séries de casos de pacientes com diagnóstico semelhante.^6 , 27^ No entanto, dada a raridade desta condição e a apresentação inespecífica, infelizmente são comuns atrasos no diagnóstico da DVOP. Presume-se que a maioria dos pacientes tenha insuficiência cardíaca congestiva esquerda devido aos achados de congestão pulmonar na TC de tórax. Alguns pacientes podem ser diagnosticados erroneamente com HPTEC devido ao defeito de perfusão incompatível na varredura V/Q. A suspeita de DVOP deve ser levantada em pacientes com histórico familiar de DVOP, histórico de doença autoimune subjacente ou exposição a medicamentos químicos/quimioterápicos. Mas especialmente naqueles com fenótipo hemodinâmico pré-capilar, hipoxemia de repouso e DLCO baixa na ausência de doenças cardíacas esquerdas e pulmonares intersticiais.

## Conclusão

A HAP com características de envolvimento venoso/capilar é uma forma incomum e rapidamente progressiva de HP. O seu diagnóstico permanece um desafio na prática clínica e a maioria dos pacientes apresenta doença avançada. Neste caso, o pronto reconhecimento clínico e o encaminhamento precoce para transplante pulmonar bilateral foram cruciais para o sucesso do caso, tendo sido possível a confirmação histopatológica do diagnóstico.
