# Supplementary material for: Hipertensão Arterial Pulmonar com Características de Envolvimento Venoso: Um Trabalho Investigativo
Source: Arq Bras Cardiol. 2024 Apr 15;121(4):e20230565. [Article in Portuguese] doi: 10.36660/abc.20230565 (PMC11081196; doi:10.36660/abc.20230565)
Supplement: Translation

# Translation: Pulmonary Arterial Hypertension with Features of Venous Involvement: A Detective's Task

**DOI:** 10.36660/abc.20230565i

**Authors:** Daniel Inácio Cazeiro, Rui Plácido, Miguel Azeredo Raposo, Joana Brito, Alexandra Borba, Tatiana Guimarães, Eugénia Pinto, Pedro Freitas, Fausto J. Pinto

**Affiliations:** 1 Lisboa Portugal Departamento de Coração e Vasos – Centro Hospitalar Universitário Lisboa Norte – Faculdade de Medicina de Lisboa – Universidade de Lisboa, Lisboa – Portugal; 2 Carnaxide Portugal Serviço de Cardiologia – Centro Hospitalar Universitário Lisboa Ocidental, Carnaxide – Portugal; 3 Lisboa Portugal Serviço de Pneumologia – Centro Hospitalar Universitário Lisboa Central, Lisboa – Portugal; 4 Lisboa Portugal Serviço de Patologia – Centro Hospitalar Universitário Lisboa Central, Lisboa – Portugal

**Keywords:** Pulmonary Arterial Hypertension, Pulmonary Veno-Oclusive Disease, Lung Transplantation, Hemangioma Capillary/surgery, Tomography-X-Ray Computed/diagnostic, imaging

## Abstract

Pulmonary veno-occlusive disease (PVOD) and pulmonary capillary hemangiomatosis are rare types of histopathological substrates within the spectrum of pulmonary arterial hypertension (PAH) with a very poor prognosis. They are characterized by a widespread fibroproliferative process of the small caliber veins and/or capillaries with sparing of the larger veins, resulting in a pre-capillary pulmonary hypertension phenotype. Clinical presentation is unspecific and similar to other PAH etiologies. Definitive diagnosis is obtained through histological analysis, although lung biopsy is not advised due to a higher risk of complications. However, some additional findings may allow a presumptive clinical diagnosis of PVOD, particularly a history of smoking, chemotherapy drug use, exposure to organic solvents (particularly trichloroethylene), low diffusing capacity for carbon monoxide (DLCO), exercise induced desaturation, and evidence of venous congestion without left heart disease on imaging, manifested by a classical triad of ground glass opacities, septal lines, and lymphadenopathies. Lung transplant is the only effective treatment, and patients should be referred at the time of diagnosis due to the rapid progression of the disease and associated poor prognosis.

We present a case of a 58-year-old man with PAH with features of venous/capillary involvement in which clinical suspicion, prompt diagnosis, and early referral for lung transplantation were determinant factors for the successful outcome.

## Introduction

### Definition, Epidemiology, and Risk Factors

Pulmonary veno-occlusive disease (PVOD) and pulmonary capillary hemangiomatosis (PCH) are considered into the spectrum of the same disease and classified as a subgroup of pulmonary arterial hypertension (PAH) due to their clinical, histopathologic, and hemodynamic similarities.

PVOD represents a minority of patients with pulmonary hypertension (PH), accounting for about 10% of PAH cases, with an estimated annual incidence of 0.1 to 0.2 cases per million.^1 , 2^However, due to the misclassification of some cases as idiopathic PAH or chronic thromboembolic PH, its true incidence may be underestimated.^3 – 5^PVOD tends to affect men and women in the same proportion,^6^although there seems to be a male predominance in idiopathic cases.^7^

Despite its unknown pathogenesis, there are several risk factors identified for PVOD. Biallelic mutations of the EIF2AK4 gene were recognized in familial cases and in about 25% of sporadic cases.^8 , 9^Exposure to some chemotherapy drugs, such as bleomycin, mitomycin, cisplatin, among others,^10 – 13^and organic solvents (mainly trichloroethylene)^7^are associated with an increased risk. Patients with PVOD, as compared with other subtypes of PAH, have greater previous smoking exposure.^6^There seems to be an association with autoimmune disorders, particularly with systemic sclerosis.^14 , 15^Several other mechanisms have been proposed but are not likely to be causative (infections, thrombotic diathesis, congenital factors, and oral contraceptives).^16^

### Histopathology

PVOD is a fibroproliferative disease that primarily affects the small pulmonary veins with relative sparing of the larger veins. There is an extensive and diffuse occlusion of the small caliber veins due to smooth muscle hypertrophy and collagen matrix deposition.^1 , 17^Long-standing venous injury may result in arterialization of pulmonary veins due to an increase in elastic fibers, often mistaken for PAH changes.^1^

Pulmonary arterioles may be concomitantly involved, with histological characteristics shared with PAH; however, arterial plexiform lesions (typical in PAH) are usually absent.^1 , 18^Alveolar capillaries may be engorged, consistent with PCH. A retrospective cohort study revealed that 73% of patients with PVOD had coexisting PCH, while most of the patients who received a diagnosis of PCH also had venous changes, postulating the hypothesis that PVOD and PCH represent the same diagnosis.^17^

Other findings include dilated pulmonary/pleural lymphatics, probably secondary to venous congestion,^18^and hemosiderin-laden alveolar macrophages, as a result of chronic congestion and hemorrhage.^19^

### Clinical evaluation and diagnosis

The definitive diagnosis of PVOD is established through histopathologic examination of lung tissue. However, biopsy is not recommended due to an increased risk of life-threatening pulmonary bleeding.^1^Therefore, histologic confirmation may not be obtained until it is discovered in an explanted lung or autopsy. Nowadays, a non-invasive diagnostic approach is recommended, based on certain clinical features and diagnostic testing results.^6^

Clinical presentation of PVOD is nonspecific and similar to other PAH etiologies.^6^Likewise, there are no significant hemodynamic differences.^6^PVOD patients have paradoxical normal wedge pressure due to the fact that the disease occurs in the small veins, without obstruction of larger pulmonary veins. Therefore, the static column of blood produced by the catheter reflects the normal pressure in the larger veins with the same caliber as the occluded vessel.^6 , 20^In other words, pulmonary arterial wedge pressure (PAWP) measures pressures in larger pulmonary veins that are unaffected by the disease, rather than the actual increased pressures in small pulmonary venules and capillaries. Nevertheless, some additional findings may raise suspicion for a diagnosis of PVOD:

– Evidence of venous congestion without significant left heart disease may be seen on chest imaging of PVOD patients, usually as diffuse ground glass opacities, septal thickening, and mediastinal lymphadenopathy.^6 , 21^In one study, when one or more of these three elements were present, there was a reported sensitivity and specificity of over 80 and 67 percent, respectively.^21^Another study with 25 patients reported the presence of at least one typical radiological characteristic in all patients and at least two radiological findings in 92% of them.^22^However, the absence or the presence of only one HRCT sign does not completely rule out the diagnosis of PVOD. If there is uncertainty regarding the diagnosis of PVOD, repeating HRCT during the disease may be helpful as it can reveal a progressive worsening of the abnormalities, especially after the initiation of treatment.
– Pulmonary function tests (PFT) and six-minute walking test (6MWT) are also very important to help distinguish between PVOD and PAH. Patients with PVOD have much lower DLCO, as well as oxygen saturation nadir on 6MWT;^6^
– Development of pulmonary edema after acute vasoreactivity testing or administration of pulmonary vasodilators, probably due to an increase in transcapillary hydrostatic pressure, which results in transudation of fluid to the alveolar space.^6 , 23 , 24^This feature is considered highly suggestive of PVOD. Patients with acute vasodilator response may develop severe pulmonary edema rapidly after initiation of calcium channel blocker therapy, and this treatment is contraindicated.

The combination of pre-capillary PH through right heart catheterization [mean pulmonary arterial pressure (mPAP) > 20 mmHg, PAWP ≤ 15 and pulmonary vascular resistance (PVR) > 2 Wood units], the above findings, and the presence of risk factors reinforce the clinical diagnosis of PVOD.^2^

### Management

Bilateral lung transplantation is currently considered the only effective therapy for PVOD.^1 , 2 , 16^Due to the rapid progression of the disease, patients should be referred at the time of diagnosis.^1 , 2 , 16^

Supportive measures include oxygen therapy, diuretics, anti-pneumococcal, influenza, and SARS-CoV-2 vaccinations, and smoking cessation. Anticoagulation is controversial – while some observational studies suggest benefit, others advise against it due to a greater frequency of occult alveolar hemorrhage.^25^

PH-specific therapy may be administered as a bridge to lung transplantation.^2^However, patients are at increased risk of developing pulmonary edema, respiratory failure, and even death.^23^When indicated, a cautious trial with a single oral agent (phosphodiesterase inhibitors or endothelin receptor antagonists) is recommended in symptomatic patients with slow up-titration and close monitoring for complications. In more severe patients, intravenous treatment with epoprostenol may also be considered.^2^

### Prognosis

The prognosis of PVOD is poor. Studies report a one-year mortality of up to 72%.^23^The average time from first symptoms to death or lung transplantation is two years.^6^

### Clinical Case

A 58-year-old man was referred to the outpatient consultation due to a history of rapidly progressive dyspnea on exertion (WHO functional class III), cough, exertional syncope, and lower limb edema for the last two months.

He had a previous diagnosis of pulmonary emphysema, with no functional impairment, attributed to smoking habits in the past (40 pack-years) and dyslipidemia. Due to his previous job as an airline technician, the patient had been exposed to several agents, such as phenolic compounds, resins, and glass fibers.

He was on a umeclidinium bromide/vilanterol inhaler (55/22 ug od), furosemide (40 mg od), and estazolam (2 mg bid). He had also been on long-term oxygen therapy for a month.

He denied any previous family history of PH.

The patient underwent several diagnostic exams:

- Laboratory studies showed erythrocytosis (hemoglobin level of 17.7 g/dL) and elevated NT-proBNP (3110 pg/mL). Basic immunology laboratory work-up, including screening tests for anti-nuclear antibodies, anti-centromere antibodies, and anti-Ro, was unremarkable.
- Electrocardiogram (ECG) revealed QRS right axis electric deviation in the frontal plane, incomplete bundle branch block and a right ventricle strain pattern.
- Transthoracic echocardiogram depicted a high probability of PH with dilated right heart chambers and impaired right ventricle systolic function. Additionally, it excluded left heart disease.
- PFT showed very low DLCO (35%) even when corrected for alveolar volume (45%) with preserved volumes and flows.
- 6MWT with a distance of 180 m and a significant effort desaturation (nadir of 79%) without supplementary oxygen.
- Computed tomography angiography (CTA) showed emphysematous changes, ground-glass opacities, and mediastinal lymphadenopathies, with no signs of thromboembolic disease ( Figure 1 ).
- Right-heart catheterization confirmed the diagnosis of a pre-capillary phenotype, with a mean pulmonary artery pressure of 45 mmHg, pulmonary capillary wedge pressure of 15 mmHg, pulmonary vascular resistance of 7.5-8.5 Wood units and cardiac index of 1.98 L/min/m2. Acute vasoreactivity test performed with inhaled nitric oxide at 30 p.p.m was negative and without complications.
- Genetic testing with a next generation sequencing panel excluded any relevant mutations, namely in the EIF2AK4 gene.

**Figure 1:** High-resolution computed tomography features of pulmonary veno-occlusive disease. Panel A – centrilobular ground-glass opacities (green arrows). Panel B - dilated pulmonary main trunk. Panel C - latero-aortic lymph node enlargement (red arrow). Panel D – presence of septal lines (blue arrows).

Given the previous smoking history, rapid progression of symptoms, very low DLCO in the absence of significant parenchymal lung disease, and radiological findings on CTA, the diagnosis of PAH with features of venous/capillary involvement (Group 1.6) was assumed. In this clinical scenario, initial sequential combination therapy with tadalafil and bosentan was pursued, and the patient was urgently referred for bilateral lung transplantation.

During follow-up, after careful up-titration of vasodilator therapy and diuretics, supplementary oxygen, and inclusion in a cardiorespiratory rehabilitation program, there was an improvement in 6MWT distance (180 ➩ 300 m), O_2_effort saturation (nadir of 92%), and NT-proBNP levels (3310 ➩ 1382 pg/mL).

The patient underwent bilateral lung transplant six months after the first appointment and nine months after symptom onset. Histopathology of the explanted lungs revealed partial/total occlusion of small caliber veins due to intimal fibrous proliferation, as well as occlusion of small caliber arteries with intimal hyperplasia, medial hypertrophy and recanalization, alveolar septa fibrosis, vascular congestion, areas of edema and hemorrhage, and pigmented macrophage desquamation in the alveolar spaces ( Figure 2 ). These findings confirmed the diagnosis of PAH with features of venous/capillary involvement.

**Figure 2:** Histology from lungs of the patient. Panel A – Hematoxilin-eosin staining depicting septal veins and pre-septal venules obliterated by collagen-rich, loose fibrosis; the parenchyma is otherwise lacking important interstitial remodelling. Panel B - Hematoxilin-eosin staining showing patchy foci of capillary haemangiomatosis in association with remodelled pulmonary vessels and microvessels (centre). Panels C and D – Verhoeff stainings showing fibrous intimal hyperplasia and hypertrophy of the muscular layer with sub-occlusion of the lumens. Scale bars: A), C) 100 μm; B) 50 μm; D) 20 μm.

The overall clinical characteristics of this case are in line with what has been published in several case series of patients with a similar diagnosis.^6 , 27^However, given the rarity of this condition and the nonspecific presentation, delays in PVOD diagnosis are unfortunately common. Most patients are usually presumed to have congestive left heart failure due to the findings of pulmonary congestion on the CT chest. Some patients may be misdiagnosed with CTEPH due to the mismatched perfusion defect from V/Q scanning. Suspicion of PVOD should be raised in patients with a history of familial PVOD, a history of underlying autoimmune disease or chemical / chemotherapeutic drug exposures. But especially in those with a hemodynamic pre-capillary phenotype, rest hypoxemia, and low DLCO in the absence of left heart and interstitial lung diseases.

## Conclusion

PAH with features of venous/capillary involvement is an uncommon and rapidly progressive form of PH. Its diagnosis remains challenging in clinical practice, with most patients presenting with advanced disease. In this case, prompt clinical recognition and early referral for bilateral lung transplantation were crucial for the patient's successful outcome, having been possible the histopathological confirmation of the diagnosis.
